# The Geometric Series Hypothesis of Leaf Area Distribution and Its Link to the Calculation of the Total Leaf Area per Shoot of *Sasaella kongosanensis* ‘Aureostriatus’

**DOI:** 10.3390/plants14010073

**Published:** 2024-12-29

**Authors:** Yong Meng, David A. Ratkowsky, Weihao Yao, Yi Heng, Peijian Shi

**Affiliations:** 1 Hunan Academy of Forestry, #658 Shaoshan South Road, Changsha 410004, China; yongmeng@hnlky.cn; 2 Co-Innovation Center for Sustainable Forestry in Southern China, Bamboo Research Institute, Nanjing Forestry University, #159 Longpan Road, Nanjing 210037, China; whyao@njfu.edu.cn (W.Y.); hengyi@njfu.edu.cn (Y.H.); 3 Tasmanian Institute of Agriculture, University of Tasmania, Private Bag 98, Hobart 7001, Australia; d.ratkowsky@utas.edu.au

**Keywords:** allometric relationship, common ratio, Montgomery equation, root-mean-square error, proportional relationship, *Sasaella kongosanensis* ‘Aureostriatus’

## Abstract

Total leaf area per shoot (*A*_T_) can reflect the photosynthetic capacity of a shoot. A prior study hypothesized that *A*_T_ is proportional to the product of the sum of the individual leaf widths per shoot (*L*_KS_) and the maximum individual leaf length per shoot (*W*_KS_), referred to as the Montgomery–Koyama–Smith equation (MKSE). However, empirical evidence does not support such a proportional relationship hypothesis, as *A*_T_ was found to allometrically scale with *L*_KS_*W*_KS_, i.e., $A_{T}\propto {\left(L_{KS}W_{KS}\right)}^{\alpha }$, where α≠1, referred to as the power law equation (PLE). Given that there is variation in the total number of leaves per shoot (*n*), little is known about whether the leaf area distribution has an explicit mathematical link with the sorted leaf area sequence per shoot, and it is unknown whether the mathematical link can affect the prediction accuracy of the MKSE and PLE. In the present study, the leaves of 500 shoots of a dwarf bamboo (*Sasaella kongosanensis* ‘Aureostriatus’) were scanned, and the leaf area, length, and width values were obtained by digitizing the leaf images. We selected the shoots with *n* ranging from 3 to 10, which accounted for 76.6% of the totally sampled shoots (388 out of 500 shoots). We used the formula for the sum of the first *j* terms (*j* ranging from 1 to *n*) of a geometric series (GS), with the mean of the quotients of any adjacent two terms (denoted as ${\bar{q}}_{A}$) per shoot as the common ratio of the GS, to fit the cumulative leaf area observations. Mean absolute percentage error (MAPE) was used to measure the goodness of fit of the GS. We found that there were 367 out of 388 shoots (94.6%) where 1 < ${\bar{q}}_{A}$ < 1.618 and MAPE < 15%, and these 367 shoots were defined as valid samples. The GS hypothesis for leaf area distribution was supported by the result that the MAPE values for most valid samples (349 out of 367, i.e., 95.1%) were smaller than 5%. Here, we provide a theoretical basis using the GS hypothesis to demonstrate the validity of the MKSE and PLE. The MAPE values for the two equations to predict *A*_T_ were smaller than 5%. This work demonstrates that the leaf area sequence per shoot follows a GS and provides a useful tool for the calculation of total leaf area per shoot, which is helpful to assess the photosynthetic capacity of plants.

## 1. Introduction

Leaves are the main photosynthetic organs of plants. The leaf area, leaf thickness, and leaf dry mass per unit area (LMA) are closely related to the photosynthetic capacity of plants [1,2,3]. Estimating the total leaf area per plant (or shoot) can then be used to evaluate the photosynthetic capacity of the plant (or shoot) [4,5,6]. Previous studies were focused on the prediction of leaf area using abiotic factors (such as the temperature, precipitation, and light availability) and biotic factors (such as the competition across and within species, plant age, and the length and width of the leaf itself) [7,8,9,10,11]. However, the leaf area distribution along the longitudinal orientation from the crown top to the crown bottom at the individual plant (or shoot) level was seldom reported. Due to the difference in light interception across different crown positions, there is a significant difference in leaf area and LMA between shade and sun leaves [1,12,13,14]. Shade leaves are larger than sun leaves, whereas the LMA values of sun leaves are greater than those of shade leaves. In addition, prior studies showed that the total leaf area per shoot is positively correlated with the number of leaves per shoot [5,6]. However, relevant studies were seldom involved in exploring the geometric and statistical features of the leaf area distribution per shoot, which are important for exploring plant strategies for resource allocation in leaves due to differences in light interception at different heights and orientations in the crown [14,15]. Huang et al. [15] quantified the variation in individual leaf areas for each of 240 shoots of *Shibataea chinensis* Nakai using the Gini coefficient, which is widely used to measure the income inequality of different economies [16], and found that the Gini coefficients for all 240 *S. chinensis* shoots had a mean of 0.141 with a standard error of 0.047, which indicated that the extent of the variation in individual leaf areas per shoot was low. However, the relationship between any two adjacent leaf areas in the sorted leaf area sequence in increasing order per shoot has been seldom studied with the exception of a recent study [17]. In the following text, we will prove that such a relationship can influence the calculation of the total leaf area per shoot.

**Figure 1:**
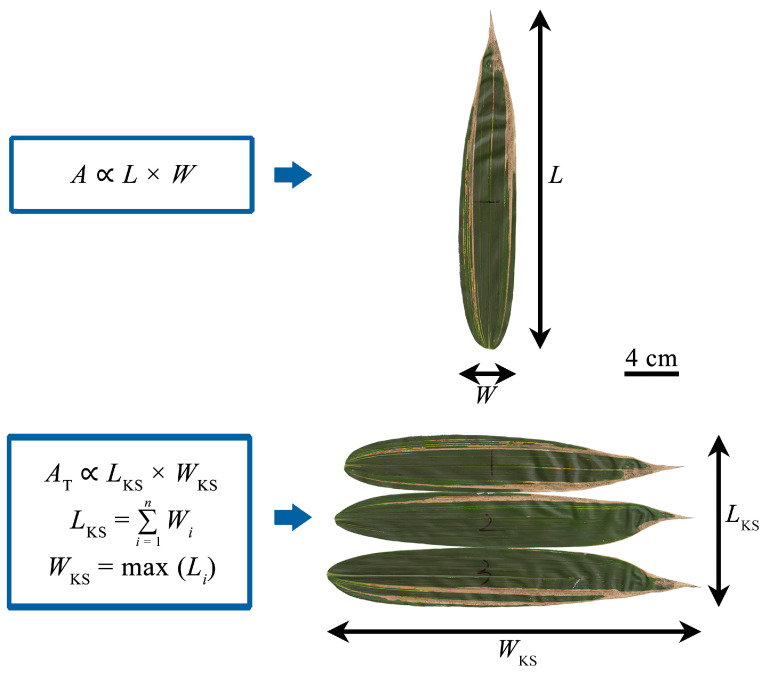
The Montgomery equation (ME) and the Montgomery–Koyama–Smith equation (MKSE) illustrated by leaves of a *Sasaella kongosanensis* ‘Aureostriatus’ shoot. The ME assumes that individual leaf area (*A*) is proportional to the product of individual leaf length (*L*) and width (*W*); the MKSE assumes that the total leaf area per shoot (*A*_T_) is proportional to the product of the sum of leaf widths per shoot (*L*_KS_) and the maximum leaf length per shoot (*W*_KS_). There are three leaves in the shoot example, i.e., *n* = 3.

Individual leaf area (*A*) has been demonstrated to be proportional to the product of individual leaf length (*L*) and width (*W*) [11,18,19,20,21,22,23,24,25,26,27,28], i.e.,

(1) A=kLW,

where *k* is the proportionality coefficient of Equation (1), and this equation is referred to as the Montgomery equation (ME). The numerical value of *k* was found to range between 1/2 and π/4 for many broad-leaved plants, and is closely related to leaf shape [11,26,27]. Koyama and Smith [5] proposed a hypothesis that the total leaf area per shoot (denoted as *A*_T_ hereinafter) is proportional to the product of the sum of the individual leaf widths per shoot (*L*_KS_) and the maximum leaf length (*W*_KS_), i.e.,

(2) $$A_{T}=k_{\mathrm{KS}}L_{\mathrm{KS}}W_{\mathrm{KS}},$$

where *k*_KS_ is the proportionality coefficient of Equation (2). This equation is referred to as the Montgomery–Koyama–Smith equation (MKSE) (Figure 1). There is a need to point out a difference between the ME and MKSE, in that the former is used to calculate the individual leaf area, and the latter is used to calculate the total leaf area per shoot (i.e., the sum of leaf areas per shoot). The validity of the MKSE has been demonstrated for seven species with different growth forms, including trees, erect herbs, rosette herbs, and dwarf bamboo [5,6]. However, empirical evidence shows that the goodness of fit of the MKSE is worse than that of a power law equation (PLE) that assumes a scaling relationship between *A*_T_ and *L*_KS_*W*_KS_, i.e.,

(3) $$A_{T}=\beta {\left(L_{\mathrm{KS}}W_{\mathrm{KS}}\right)}^{\alpha },$$

where β is the normalized constant, and α is the scaling exponent of *A*_T_ vs. *L*_KS_*W*_KS_. The numerical value of α was found to be significantly smaller than unity for the studied plants [5,6]. The deviation of α from unity was not explained by prior work for the leaf material. Yan et al. [29] compared the MKSE and PLE in estimating the total stomatal area in each of 720 stomatal micrographs using 12 Magnoliaceae species and provided a theoretical explanation of why the numerical value of α was smaller than unity. They derived the conditions under which the MKSE holds true, including: (i) there is a proportional relationship between *A* and *LW*, as the ME assumes, (ii) the stomatal area sequence in increasing order for each micrograph follows a geometric series with the same common ratio, (iii) stomatal width is proportional to stomatal length, and (iv) the number of stomata per micrograph is a constant across micrographs. Prior studies have demonstrated that the first condition holds true for broad-leaved plants [11,26,27]. If the other conditions are violated, *A*_T_ tends to allometrically (rather than isometrically) scale with *L*_KS_*W*_KS_. However, for the leaf area distribution per shoot, whether the sorted leaf area sequence per shoot follows a geometric series, the same as the stomatal area sequence per micrograph, has been seldom tested with the exception of a recent study [17]. In addition, prior studies showed that *W* does not tend to isometrically scale with individual length [26,27]. If individual leaf width allometrically scales with *L*, there is a need to test whether the MKSE still holds true when the other conditions are met. If the scaling relationship between *W* and *L* does not affect the validity of the MKSE, whether violation of the fourth condition, i.e., the number of leaves per shoot is not a constant among shoots, can lead to an allometric relationship between *A*_T_ and *L*_KS_*W*_KS_ also merits investigation.

Wang et al. [6] showed that, with increasing number of leaves per shoot, the extent of the variation in leaf area per shoot increases due to self-shading across leaves. Such variation is likely to violate the geometric series hypothesis of leaf area distribution. Severe self-shading can lead to the abnormal development of several leaves and produce too large leaves [30]. To avoid this problem, we selected *Sasaella kongosanensis* ‘Aureostriatus’, a Poaceae species, as the plant material because the leaves of this species are sparsely aggregated at the top (distal) internodes of shoots and thus have a very low extent of self-shading.

In the present study, we used the leaves of 367 *S. kongosanensis* ‘Aureostriatus’ shoots (selected from the 500 shoot samples) to test: (i) whether the leaf area sequence per shoot follows a geometric series, (ii) whether leaf width allometrically scales with leaf length, and (iii) whether the MKSE still holds true under the condition that the two hypotheses are validated.

## 2. Theory

Let us assume that *W* scales with *L*, which can be expressed by a power law equation, and such a power law equation is valid for describing a scaling relationship between two interdependent biological variables [31]:

(4) $$W=bL^{a},$$

where *b* is the normalized constant, and *a* is the scaling exponent of *W* vs. *L*. If *a* = 1, *W* isometrically scales with *L*; if *a* ≠ 1, *W* allometrically scales with *L*. It is apparent that *a* and *b* are greater than zero. Let *n* denote the number of leaves per shoot. Assume that the leaf length sequence of a shoot is sorted in increasing order, i.e., $L_{1}<L_{2}<\cdots <L_{n}$. According to the definitions of *A*_T_, *L*_KS_, and *W*_KS_ (Figure 1), the Montgomery equation (i.e., Equation (1)), and Equation (4), we have the following relationships:

(5) $$A_{T}=\sum_{i=1}^{n}A_{i}=\sum_{i=1}^{n}\left(kL_{i}W_{i}\right)=kb\sum_{i=1}^{n}L_{i}^{a+1},$$

(6) $$L_{\mathrm{KS}}=\sum_{i=1}^{n}W_{i}=b\sum_{i=1}^{n}L_{i}^{a},$$

and

(7) $$W_{\mathrm{KS}}=\max\left\{L_{i}\right\}=L_{n}.$$

Then $A_{T}/\left(L_{KS}W_{KS}\right)=k\sum_{i=1}^{n}L_{i}^{a+1}/\sum_{i=1}^{n}\left(L_{i}^{a}L_{n}\right)$. Let *q* represent the common ratio of the leaf length sequence in increasing order, which is assumed to follow a geometric series (*q* > 1). According to the general formula of the geometric series, $L_{n}=L_{i}q^{n-i}$ (1≤i≤n). Let us define a function $w\left(q,n\right)$ as follows:

(8) $$w\left(q,n\right)=\frac{1}{k}\frac{A_{T}}{L_{\mathrm{KS}}W_{\mathrm{KS}}}=\frac{\sum_{i=1}^{n}L_{i}^{a+1}}{\sum_{i=1}^{n}\left(L_{i}^{a}L_{n}\right)}=\frac{\sum_{i=1}^{n}L_{i}^{a+1}}{\sum_{i=1}^{n}\left(L_{i}^{a+1}q^{n-i}\right)}.$$

Because $q^{n-i}\geq 1$ (the equality is tenable only when *i = n*), $w\left(q,n\right)<1$. It is apparent that

(9) $$L_{i}^{a+1}=L_{1}^{a+1}q^{(a+1)\left(i-1\right)}.$$

After substituting Equation (9) into Equation (8), we have the following relationship:

(10) $$w\left(q,n\right)=\frac{L_{1}^{a+1}\sum_{i=1}^{n}q^{\left(a+1\right)\left(i-1\right)}}{L_{1}^{a+1}\sum_{i=1}^{n}q^{\left(a+1\right)\left(i-1\right)+\left(n-i\right)}}=\frac{\sum_{i=1}^{n}q^{ai+i}}{\sum_{i=1}^{n}q^{ai+n}}.$$

**Figure 2:**
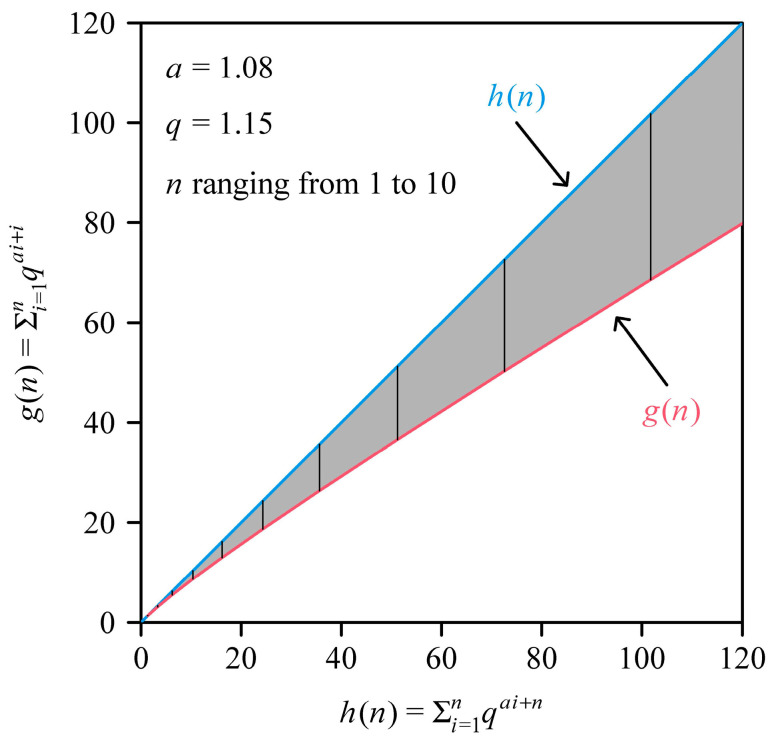
Comparison between $g\left(n\right)$ (red curve) and $h\left(n\right)$ (blue 45° straight line). The distribution of the leaf length sequence of a shoot is assumed to follow a geometric series with the common ratio *q* = 1.15; *a* represents the scaling exponent of individual leaf width vs. individual leaf length; the numerical value of *n* ranges from 1 to 10, and the corresponding $g\left(n\right)$ and $h\left(n\right)$ values are the endpoints of the vertical segments from the left to the right. It is apparent that $g\left(n\right)/h\left(n\right)<1$ is a decreasing function of *n*.

Equation (10) suggests that $A_{T}/\left(L_{KS}W_{KS}\right)$ is determined by *q* and *n*, irrespective of the smallest leaf length per shoot (i.e., *L*_1_). If the common ratio of the leaf length sequence (*q*) and the number of leaves per shoot (*n*) are both constant across different shoots, $w\left(q,n\right)$ is a constant that is smaller than unity according to Equation (10). In that case, $A_{T}/\left(L_{KS}W_{KS}\right)=k\cdot w\left(q,n\right)$ is also a constant that is smaller than unity considering *k* < 1 (see Equation (1)). Given that there is usually a very small variation in *q* across shoots, let us assume *q* is a constant in Equation (10) for all shoots of the same species. Then $w\left(q,n\right)=w\left(n\right)$, which is apparently a decreasing function of *n* given *i* ≤ *n* in Equation (10). Let $g\left(n\right)=\sum_{i=1}^{n}q^{ai+i}$ and $h\left(n\right)=\sum_{i=1}^{n}q^{ai+n}$. Since $g\left(n\right)$ and $h\left(n\right)$ are both increasing functions of *n*, and $w\left(n\right)=g\left(n\right)/h\left(n\right)<1$ is a decreasing function of *n*, it means that increases in $g\left(n\right)$ do not keep pace with increases in $h\left(n\right)$ (supposing $g\left(n\right)$ is plotted against $h\left(n\right)$; Figure 2). This means that the scaling exponent of $g\left(n\right)$ vs. $h\left(n\right)$ should be smaller than unity. Because $A_{T}\propto g\left(n\right)$ and $L_{KS}W_{KS}\propto h\left(n\right)$, the numerical value of α in Equation (3) should be also smaller than unity under the condition that *n* varies across different shoots. In the above proofs, the numerical value of *a* does not affect the above derivations. Yan et al. [29] gave similar derivations when *a* = 1 (see their Discussion section and Figure 9 therein), i.e., the proofs for a special case of the present study with *a* = 1. The present study relaxes the limit that leaf (stomatal) width should be proportional to leaf (stomatal) length for rendering the MKSE to hold true, which was set in [5,29]. The present study predicts an allometric relationship between $A_{T}$ and $L_{KS}W_{KS}$ due to the variation in *n* across different shoots.

## 3. Materials and Methods

### 3.1. Leaf Sampling and Data Acquisition

In March 2023, 500 *S. kongosanensis* ‘Aureostriatus’ shoots were sampled from Nanjing Forestry University Xinzhuang Campus (118°48′58″ E, 32°4′37″ N). At the sampling site, *S. kongosanensis* ‘Aureostriatus’ individuals were mixed with *Pleioblastus argenteostriatus* individuals. However, *S. kongosanensis* ‘Aureostriatus’ shoots were taller than *P. argenteostriatus* shoots, and the leaves of *S. kongosanensis* ‘Aureostriatus’ were generally unshaded by neighboring species. The interannual mean daily temperature was 15.6 °C, the interannual mean cumulative precipitation was 1058 mm, the interannual mean relative humidity was 75.7%, and interannual mean sunshine duration was 2038 h for Nanjing, in which the study site is located, based on the climatic data between 1951 and 2012 [32]. The soil type in the study site is predominantly mountain yellow-brown and grey-brown soil [33].

We harvested all leaves of each of the 500 shoots, removed the pseudo-petioles of leaves, and then scanned the leaves with a photo scanner (V550, Epson Indonesia, Batam, Indonesia) at 600 dpi resolution. The scanned images were converted to black and white bitmap images at 600 dpi using Adobe Photoshop (version 13.0; Adobe Systems Incorporated, San Jose, CA, USA). The MATLAB (version ≥ 2009a; MathWorks, Natick, MA, USA) procedure developed in [34,35] was used to extract the planar coordinates of each leaf boundary, and the “bilat” function in the “biogeom” package (version 1.3.5) [36] based on R software (version 4.2.0) [37] was used to calculate the leaf area (*A*), length (*L*), and width (*W*) of each of 2111 leaves from the 500 *S. kongosanensis* ‘Aureostriatus’ shoots.

The number of leaves per shoot (*n*) of the harvested 500 shoots ranged from 1 to 19. There were 100 shoots with *n* = 1 and 2, accounting for 20% of the total shoot samples, which are apparently meaningless to test the validity of the geometric series hypothesis for the leaf area sequence. In that case, the geometric series hypothesis for the leaf area sequence per shoot for these shoots with one leaf or two leaves apparently held true. It was impossible to test the prediction errors for those shoots. We also excluded the shoot groups with *n* ranging from 11 to 19, accounting only for 2.4% of the total shoot samples, because the number of shoots (from 1 to 4 shoots) for each group was too small, which was likely to represent abnormal leaf development. Thus, we only kept the remaining 388 shoots with *n* ranging from 3 to 10, which accounted for 76.6% of the totally sampled shoots (Table 1).

### 3.2. Data Analysis

To test the validity of the geometric series hypothesis for the leaf area distribution per shoot, we sorted the leaf areas for each shoot and used the formula for the sum of the first *j* terms (*j* = 1, 2, 3, …, *n*, where *n* represents the number of leaves of a shoot) of a geometric series (denoted as *S_j_*) to fit the observed cumulative leaf area for the first *j* leaves of the sorted leaf area sequence for each shoot, as follows:

(11) $$S_{j}=\sum_{u=1}^{j}A_{u}=A_{1}\frac{{\left(1-q_{A}\right)}^{j}}{1-q_{A}},$$

where *A*_1_ represents the smallest leaf area, *q_A_* represents the common ratio of this geometric series. In the present study, *q_A_* was estimated using the mean of the quotients of any two adjacent leaf areas of a shoot (denoted as ${\bar{q}}_{A}$) [17], i.e.,

(12) $${\bar{q}}_{A}=\frac{\sum_{i=2}^{n}\left(A_{i}/A_{i-1}\right)}{n-1}.$$

After substituting Equation (12) into Equation (11), we could calculate the sum of the *j* terms of the geometric series. However, the probable abnormal value of *A*_1_ can lead to a large prediction error in Equation (11). To solve this problem, we used the mean of the predicted *A*_1_ values based on all terms in the geometric series, using the general term formula of the geometric series $A_{i}=A_{1}q_{A}^{i-1}$, to estimate *S_j_* (denoted as ${\bar{S}}_{j}$) [17] as follows:

(13) $${\bar{S}}_{j}=\left\{\frac{1}{n}\sum_{i=1}^{n}\left(A_{i}/{\left({\bar{q}}_{A}\right)}^{i-1}\right)\right\}\frac{{\left(1-{\bar{q}}_{A}\right)}^{j}}{1-{\bar{q}}_{A}}.$$

We calculated the mean absolute percentage error (MAPE) between the observed and predicted cumulative leaf areas for each shoot to reflect the validity of the geometric series hypothesis [17], as follows:

(14) $$\mathrm{MAPE}=\frac{1}{n}\sum_{j=1}^{n}\left\{\left|{\bar{S}}_{j}-\sum_{u=1}^{j}A_{u}\right|/\sum_{u=1}^{j}A_{u}\right\}\times 100\%.$$

As a rule of thumb, MAPE < 5% indicates a good fit [27,28]. In the present study, we excluded the shoots with ${\bar{q}}_{A}$ ≥ 1.618 or MAPE ≥ 15% which represented individuals with leaves that were abnormally developed or were partially damaged. We restricted the upper limit of *q_A_* using an analogous rule of the triangle inequality theorem, i.e., the sum of the lengths of the two smaller sides is larger than the length of the largest side, and suggested that the sum of the areas of the two smaller leaves was larger than the area of the largest leaf among three adjacent leaves (sorted by their leaf areas in increasing order). That is, $A_{i}<A_{i-1}+A_{i-2}$ (*i* > 3), which is equivalent to $L_{1}q_{A}^{i-1}<L_{1}q_{A}^{i-2}+L_{1}q_{A}^{i-2}$ ⟺ $q_{A}^{2}-q_{A}-1<0$ ⇒ $q_{A}<\left(1+\sqrt{5}\right)/2\approx 1.618$. We named the remaining dataset with ${\bar{q}}_{A}$ < 1.618 and MAPE < 15% “the valid dataset” and used it to carry out the following analyses.

To test the validity of the Montgomery equation (ME), we used ordinary least-squares to fit Equation (1). To stabilize the variation in *A*, before carrying out the linear regression, both sides of Equation (1) were log-transformed as follows:

(15) $$\log\left(A\right)=\log\left(k\right)+\log\left(LW\right),$$

where log(*A*) was regarded as the response variable, log(*LW*) was regarded as the predictor variable, and log(*k*) was regarded as the intercept.

The log-transformed form was also applied to fitting the relationships between *W* and *L* in Equation (4), and between $A_{T}$ and $L_{KS}W_{KS}$ in Equations (2) and (3), as follows:

(16) $$\log\left(W\right)=\log\left(b\right)+a\log\left(L\right),$$

(17) $$\log\left(A_{T}\right)=\log\left(k_{\mathrm{KS}}\right)+\log\left(L_{\mathrm{KS}}W_{\mathrm{KS}}\right),$$

and

(18) $$\log\left(A_{T}\right)=\log\left(\beta \right)+\alpha \log\left(L_{\mathrm{KS}}W_{\mathrm{KS}}\right).$$

The root-mean-square error (RMSE) was used to reflect the goodness of fit of Equations (15)–(18), which is expressed as follows:

(19) $$\mathrm{RMSE}=\sqrt{\frac{1}{N}\sum_{i=1}^{N}{\left({\hat{y}}_{i}-y_{i}\right)}^{2}},$$

where ${\hat{y}}_{i}$ and $y_{i}$ represent the predicted and observed response variables in Equations (15)–(18), respectively; *N* is the sample size, i.e., the number of shoots, whereas *n* in Equations (11)−(14) represents the number of leaves per shoot. As a rule of thumb, RMSE < 0.05 indicates a good fit. To test whether introducing an additional parameter was worthwhile when comparing Equations (17) and (18), i.e., the Montgomery–Koyama–Smith equation (MKSE) and power law equation (PLE), the percentage error (PE) between their RMSE values was calculated, as follows:

(20) $$\mathrm{PE}=\frac{{\mathrm{RMSE}}_{\mathrm{MKSE}}-{\mathrm{RMSE}}_{\mathrm{PLE}}}{{\mathrm{RMSE}}_{\mathrm{MKSE}}}\times 100\%,$$

where the subscript corresponds to the MKSE or PLE. As a rule of thumb, PE < 5% indicates that introducing an additional parameter is unnecessary. In addition, we also calculated the MAPE (i.e., $\frac{1}{N}\sum_{i=1}^{N}\left(\left|{{\hat{A}}_{T,i}-A}_{T,i}\right|/A_{T,i}\right)\times 100\%$) values between the observed and predicted $A_{T}$ values using the MKSE and PLE.

The bootstrap percentile method [38,39], employing 3000 bootstrapping replicates, was used to calculate the 95% confidence intervals (CIs) of the intercepts and slopes.

In addition, analysis of variance was used to test the significance of the differences in the mean common ratios among the eight shoot groups. The test for Pearson’s product moment correlation coefficient was carried out to test whether the mean of the mean common ratios for each shoot group was correlated with the number of leaves per shoot. For each shoot, there was one mean common ratio; for each shoot group with many shoots, we performed a linear regression of the mean of the mean common ratios vs. the number of leaves per shoot.

All calculations were performed and figures were constructed using software R (version 4.2.0) [37].

## 4. Results

**Figure 3:**
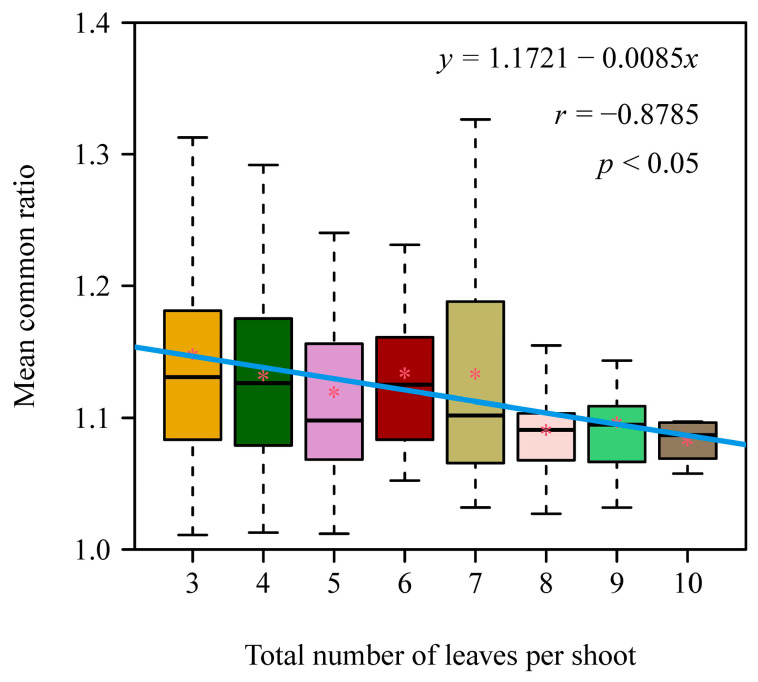
Comparison of the mean common ratios among the eight *Sasaella kongosanensis* ‘Aureostriatus’ shoot groups, and the correlation between the mean of the mean common ratios for each shoot group (*y*) and the number of leaves per shoot (*x*). Here, the horizontal solid lines in each box represent the medians; the asterisks near the medians represent the means; the whiskers extend to the most extreme data point, which is no more than 1.5 times the interquartile range from the box; the blue straight line is the regression line for the mean of the mean common ratios of each shoot group vs. the number of leaves per shoot. The *p*-value is for Pearson’s product moment correlation coefficient between *x* and *y*.

**Figure 4:**
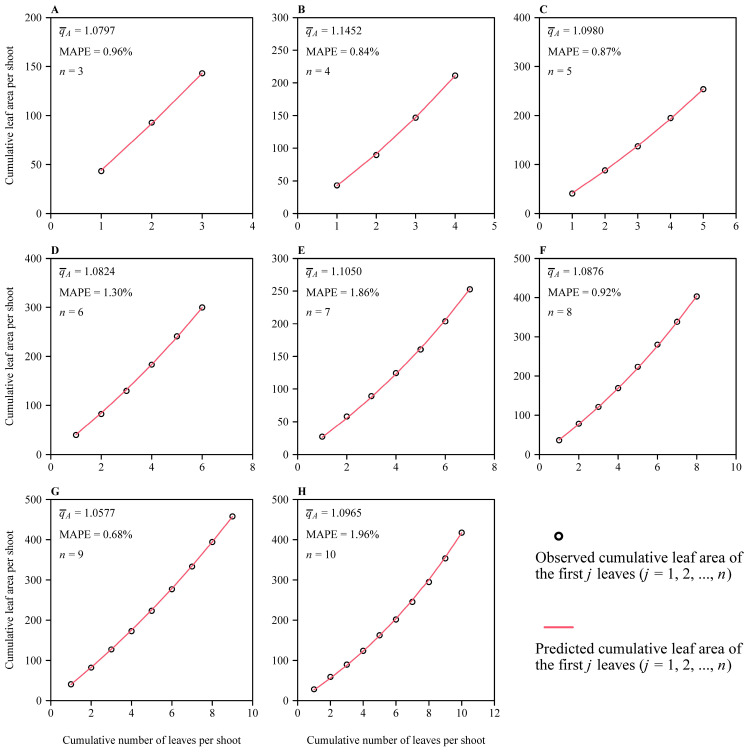
Fitted results of the geometric series to the observed cumulative leaf area sequences of eight shoot examples corresponding to the eight *Sasaella kongosanensis* ‘Aureostriatus’ shoot groups ranging from 3 to 10 (see Table 1 for details). Panels (**A**–**H**) represent different shoot groups. In each panel, ${\bar{q}}_{A}$ represents the mean common ratio of the leaf area geometric series for each shoot; MAPE is the mean absolute percentage error between the observed and predicted cumulative leaf area sequences for each shoot; *n* is the number of leaves for each shoot.

There were 367 out of 388 shoots with the number of leaves ranging from 3 to 10, which met the requirements that 1 < ${\bar{q}}_{A}$ < 1.618 and MAPE < 15% (Table S1 in the online Supplementary Material). That is, 94.6% of the selected 388 shoots were the valid dataset, in which the MAPE values ranged from 0.02% to 14.65% with a mean (±SD) of 1.78% ± 2.02%, and the ${\bar{q}}_{A}$ values ranged from 1.0110 to 1.5058 with a mean (±SD) of 1.1348 ± 0.0811. There was a significant difference in ${\bar{q}}_{A}$ values across the eight shoot groups (*F*_7, 359_ = 2.374, and *p* = 0.0221 < 0.05), and there was a significantly negative correlation between the mean of the ${\bar{q}}_{A}$ values and the number of leaves per shoot (*r* = −0.8785; *p* < 0.05) (Figure 3). Figure 4 exhibits eight examples of the geometric series fit corresponding to the eight shoot groups.

**Figure 5:**
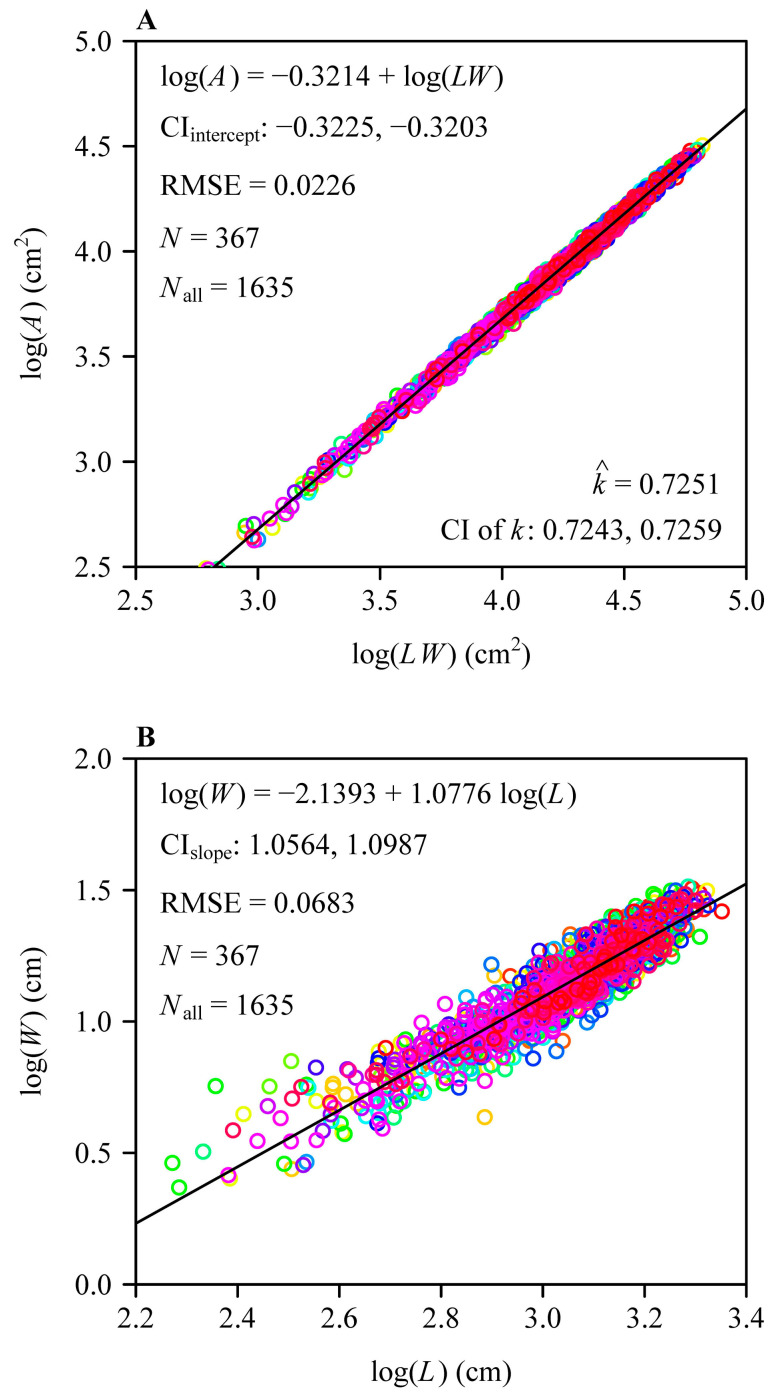
Fitted results for the proportional relationship between leaf area and the product of leaf length and leaf width (**A**), and the scaling relationship between leaf width and leaf length on a log-log scale for *Sasaella kongosanensis* ‘Aureostriatus’ (**B**). The open circles represent the observations, and the straight lines represent the regression lines; different colors represent different shoots; *N* represents the number of shoots; and *N*_all_ represents the total number of leaves for the 367 shoots.

The Montgomery equation (ME) was validated in describing the proportional relationship between leaf area (*A*) and the production of leaf length and width (*LW*), and the RMSE value was equal to 0.0226 (Figure 5A). The proportionality coefficient of the ME was estimated to be 0.7251 with 95% confidence intervals (CI) of 0.7243 and 0.7259. There was a significant allometric relationship between leaf width (*W*) and length (*L*) because the lower bound of the 95% CI of the scaling exponent of *W* vs. *L* was equal to 1.0564, which was greater than unity (Figure 5B). This meant that increases in *L* did not keep pace with increases in *W*. This confirmed our hypothesis that *W* allometrically scales with *L*.

**Figure 6:**
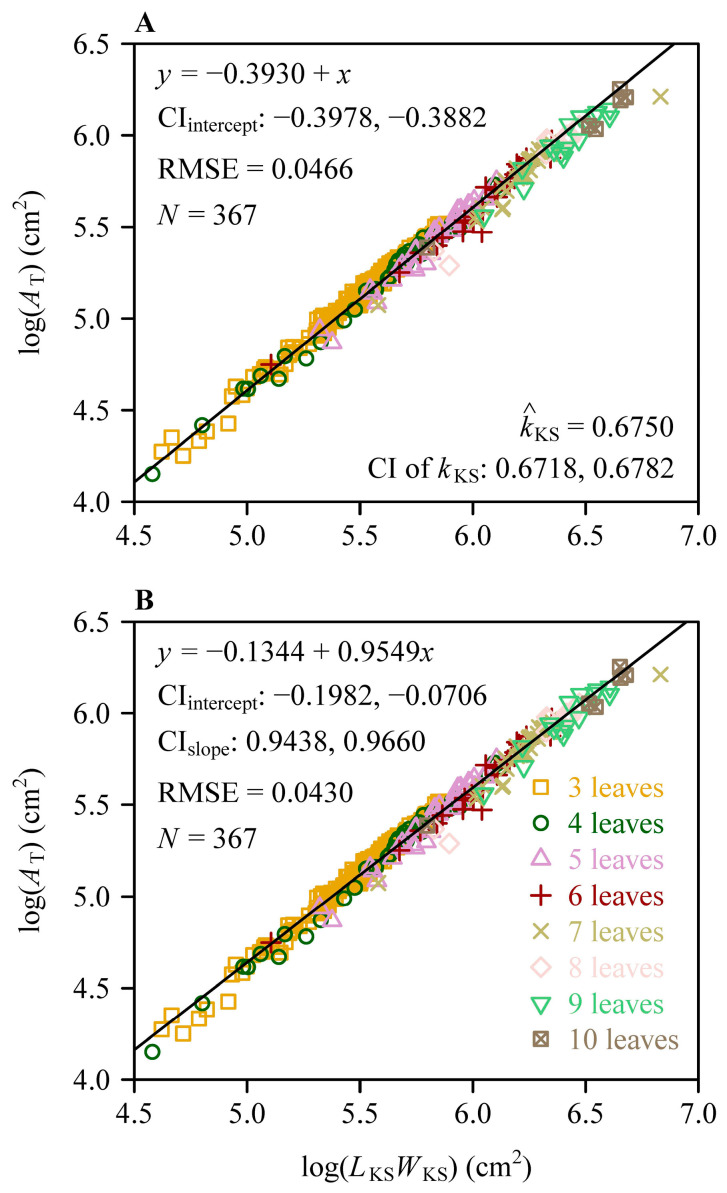
Results of fitting the Montgomery–Koyama–Smith equation (**A**) and the power law equation (**B**) between the total leaf area per shoot (*A*_T_) and the product of the sum of leaf widths and the maximum leaf length per shoot (*L*_KS_*W*_KS_) on a log-log scale for the eight *Sasaella kongosanensis* ‘Aureostriatus’ shoot groups with 3 to 10 leaves per shoot. Different symbols are the observations of different shoot groups converted on a log-log scale; CI_intercept_ is the 95% confidence interval of the intercept; CI_slope_ is the 95% confidence interval of the slope; RMSE is the root-mean-square error of the linear fitting; and *N* is the total number of shoots of the eight shoot groups. In panel (**A**), $\hat{k}$_KS_ represents the estimated value of the proportionality coefficient of the MKSE, and CI of $k_{KS}$ represents the 95% confidence interval of the proportionality coefficient of the MKSE.

The Montgomery–Koyama–Smith equation (MKSE) and power law equation (PLE) were found to be valid in describing the scaling relationship between the total leaf area per shoot (*A*_T_) and the product of the sum of leaf widths per shoot (*L*_KS_) and the maximum leaf length per shoot (*W*_KS_), reflected by two RMSE values < 0.05 (Figure 6). The estimated value of the proportionality coefficient of the MKSE was equal to 0.6750 with 95% CIs of 0.6718 and 0.6782. The RMSE value of the PLE was smaller than that of the MKSE (0.0430 vs. 0.0466), and the percentage error (PE) between the two RMSE values was 7.74% > 5%. This indicated that *A*_T_ tends to allometrically (rather than isometrically) scale with *L*_KS_*W*_KS_. Nevertheless, the mean absolute percentage error (MAPE) values between the observed and predicted *A*_T_ values by the MKSE and PLE were equal to 3.59% and 3.25%, respectively, both smaller than 5%. This meant that the two equations both provided robust predictions for 367 *A*_T_ values.

## 5. Discussion

In the present study, we excluded the shoot groups with *n* > 10, and the total number of those shoots only accounted for 2.4% of the 500 shoots. The larger the number of leaves per shoot (i.e., *n*), the larger the variation in leaf area [6]. The “diminution on ramification” rule states that “the greater the ramification, the smaller become the branches and their appendages” [40,41]. More than half of the individuals of *S. kongosanensis* ‘Aureostriatus’ had two to three leaves, which accounted for 57.2% of the totally sampled shoots (Table 1). The mean leaf area (or the mean leaf dry mass) decreases with the leafing intensity, which is defined by *n*/total above-ground non-leaf dry mass or *n*/shoot height [42,43,44,45,46], but the coefficient of variation in area across individual leaves per shoot increases with increasing *n* [6]. In general, the regularity in size distribution is likely to validate the geometric series hypothesis for a size sequence [29]. Overly ramified branches of a small amount of *S. kongosanensis* ‘Aureostriatus’ shoots produced more leaves with a large variation in leaf area. As a smaller branch tends to produce very small leaves, this led the leaf area sequence to violate the geometric series hypothesis. During the data analyses, we tried to combine the shoot groups with *n* > 10 and found that they did not largely affect the validity of the MKSE and PLE. However, those shoot groups predicted overly large mean common ratios of leaf area sequences and large prediction errors using the geometric series fit. For the selected shoot groups with *n* ranging from 3 to 10, there were still a small proportion of shoots that predicted excessively large mean common ratios and large prediction errors using the geometric series fit, which accounted for 5.4% of the 388 shoots. We checked the scanned leaves of those shoots with abnormal ${\bar{q}}_{A}$ and MAPE values, and found that the abnormal values were caused by very small leaves that newly appeared in the early spring in spite of the fact that there were no new leaves for most shoots (accounting for 94.6% of the 388 shoots). Thus, it was reasonable for us to also exclude those shoots during the analysis.

For the valid dataset (i.e., 367 shoots), the MAPE values (Equation (14) ranged from 0.02% to 14.65% with a mean (±SD) of 1.78% ± 2.02%, and the data indicated a very good fit based on the geometric series hypothesis. This should result from a low extent of self-shading across individual leaves due to the above-ground architecture of *S. kongosanensis* ‘Aureostriatus’. Most individuals only had one ramified branch with two to five leaves, which did not shade each other. However, for some larger bamboo, such as moso bamboo, there are many branches that are vertically distributed along the culm [47], and the leaves on the upper branches or those from other culms can seriously shade the leaves on the lower branches, which can lead to a large variation in leaf area [30]. In this case, the geometric series hypothesis for the leaf area distribution per shoot may not be valid. However, it deserves further testing in future studies to examine whether the number of leaves per shoot is negatively correlated with the goodness of fit of the geometric series hypothesis for the leaf area sequence per shoot using more species.

Some studies assumed that individual leaf area (*A*) is proportional to *L*^2^ (e.g., [24,48]). However, the validity of the $A\propto L^{2}$ hypothesis depends on the scaling relationship between *W* and *L*. Prior studies showed that the prediction accuracy of using *L*^2^ to predict *A* is determined by the extent of variation in the ratio of *W* to *L*, with a lower variation in *W/L* corresponding to a higher prediction accuracy using the $A\propto L^{2}$ hypothesis [26,27,49]. The present study confirmed that individual leaf width (*W*) allometrically scales with individual leaf length (*L*). Thus, using the product of two leaf one-dimensional measures (i.e., *LW*) to estimate *A* is better than only using one leaf one-dimensional measure squared (i.e., *L*^2^ or *W*^2^). In fact, the Montgomery equation (ME) that assumes a proportional relationship between *A* and *LW* has been validated by empirical evidence from different plant groups [11,18,19,20,21,22,23,24,25,26,27,28]. For bamboo taxa, Shi et al. [49] measured *A*, *L*, and *W* of 10,045 leaves from 101 bamboo taxa (subfamily Bambusoideae) with similar elongated leaf shapes and confirmed the validity of the ME for describing *A* in this subfamily. The estimated proportionality coefficients of the ME of those plants ranged from 0.62 to 0.78. The current estimated proportionality coefficient of the ME for the 1635 leaves from the 367 *S. kongosanensis* ‘Aureostriatus’ shoots was equal to 0.7251, falling within the previously reported range. The allometric relationship between *W* and *L* can account for why the ME is better than the $A\propto L^{2}$ hypothesis in calculating *A*.

The Montgomery–Koyama–Smith equation (MKSE) is actually an important extension of the ME from the individual leaf area scale to the total leaf area per shoot scale, which regards the leaves of a shoot as the leaflets of a pinnately compound leaf. Schrader et al. [11] confirmed the validity of the ME for calculating the area of pinnately compound leaves. However, refs. [5,29] provided a proposition that the MKSE holds true that leaf (stomatal) width should be proportional to leaf (stomatal) length. However, the present study relaxed such a limit. Even though they scale allometrically with each other, the MKSE still holds true. However, the present study also found that the scaling exponent of *A*_T_ vs. *L*_KS_*W*_KS_ was significantly smaller than unity, which was in accord with values reported in prior reports [5,6,29]. The scaling exponent < 1 results from the variation in the number of leaves per shoot across different shoots. Nevertheless, because of the small variation in leaf area per shoot, to assume that *A*_T_ is proportional to *L*_KS_*W*_KS_, i.e., the scaling exponent is equal to unity, was still generally valid in determining *A*_T_, which led to a prediction error of <4% (reflected by MAPE = 3.59%).

In addition, there is a need to note the difficulty and limitation in applying the ME and MKSE equations to calculate individual leaf area (*A*) and total leaf area per shoot (*A*_T_) in practice. Firstly, complex leaf shapes might pose a challenge to accurately define individual leaf length (*L*) and individual leaf width (*W*). Secondly, if the number of leaves per shoot is too large, e.g., ≥30 leaves per shoot, it will be time-consuming to measure the *L* and *W* data of a large sample of shoots (e.g., ≥120 shoots) for fitting the MKSE or PLE in a more robust manner. It is apparent that carrying out MKSE fitting requires calculations of the sum of all individual widths (*L*_KS_) and the maximum leaf length (*W*_KS_) per shoot. However, in practice, it is somewhat difficult to determine which leaf is the longest by eye. Therefore, in the field, the determination of *W*_KS_ probably deviates from the definition in [5]. Thirdly, the MKSE only applies to shoots, herbs, and shrubs with a limited number of leaves and some trees with simple branching patterns. It probably does not apply to the calculation of the total leaf area for the trees with complex branching patterns because the variation in branching patterns across individual trees tends to invalidate the similarity hypothesis of leaf arrangement in space [50]. This merits investigation in the future.

## 6. Conclusions

The leaf area sequences of 367 *S. kongosanensis* ‘Aureostriatus’ shoots were fitted to test the validity of the geometric series hypothesis for leaf area distribution per shoot, and 95.1% of the valid samples were found to follow a geometric series well. The calculated mean common ratios per shoot ranged from 1.0110 to 1.5058 with a mean (±SD) of 1.1348 ± 0.0811. The mean of those mean common ratios was found to be negatively correlated with the number of leaves per shoot. In addition, individual leaf width was found to allometrically scale with individual length; however, such an allometry did not affect the validity of the proportional relationship hypothesis between the total leaf area (*A*_T_) and the product of the sum of leaf widths per shoot (*L*_KS_) and the maximum leaf length per shoot (*W*_KS_), as the Montgomery–Koyama–Smith equation (MKSE) hypothesizes. The theoretical derivation demonstrates that variation in the number of leaves per shoot can lead the scaling exponent of *A*_T_ vs. *L*_KS_*W*_KS_ to be smaller than unity, which means that *A*_T_ tends to have an allometric relationship (rather than an isometric relationship) with *L*_KS_*W*_KS_. However, due to a small variation in the number of leaves per shoot from 3 to 10, the geometric series appears to be valid for describing the leaf area distribution per shoot, and the MKSE on average led to a prediction error < 4%. The present work provides a useful tool for calculating the total leaf area per shoot.

## Figures and Tables

**Table 1:** Frequency distribution of the number of shoots of the 500 shoots of *Sasaella kongosanensis* ‘Aureostriatus’.

*n*	*N*_E_	*N*_V_	Percentage
1	4	−	−
2	96	−	−
3	190	188	98.95%
4	52	48	92.31%
5	41	37	90.24%
6	29	27	93.10%
7	33	32	96.97%
8	17	13	76.47%
9	20	16	80.00%
10	6	6	100%
11	4	−	−
12	5	−	−
13	1	−	−
15	1	−	−
19	1	−	−

Here, *n* represents the shoot group number, which was defined according to the number of leaves per shoot; *N*_E_ represents the entire number of shoots for each shoot group; and *N*_V_ represents the valid number of shoots for each shoot group, with the calculated mean common ratio of the leaf area sequence per shoot ranging from 1 to 1.618 and the mean absolute percentage error <15% for a geometric series fit (see below for details). Percentage is equal to *N*_V_*/N*_E_ × 100%.

## Data Availability

The data can be found in the online Supplementary Table S1.

## Supplementary Materials

The following supporting information can be downloaded at: https://www.mdpi.com/article/10.3390/plants14010073/s1, Table S1: Fitted results of the geometric series to the leaf area sequences of 388 *Sasaella kongosanensis* ‘Aureostriatus’ shoots with 3 to 10 leaves for each shoot.
